# An RNA-immunoprecipitation via CRISPR/dCas13 reveals an interaction between the SARS-CoV-2 5'UTR RNA and the process of human lipid metabolism

**DOI:** 10.1038/s41598-023-36680-6

**Published:** 2023-06-27

**Authors:** Yurika Shimizu, Srinivas Bandaru, Mari Hara, Sonny Young, Toshikazu Sano, Kaya Usami, Yuta Kurano, Suni Lee, Naoko Kumagai-Takei, Shogo Takashiba, Shunji Sano, Tatsuo Ito

**Affiliations:** 1 Department of Hygiene, Kawasaki Medical School, 577 Matsushima, Kurashiki, Okayama 701-0192 Japan; 2 Department of Pathophysiology - Periodontal Science, Okayama University Graduate School of Medicine, Dentistry and Pharmaceutical Sciences, Okayama, Okayama 700-8525 Japan; 3 Department of Surgery, Division of Pediatric Cardiothoracic Surgery, University of California San Francisco, San Francisco, CA USA; 4 Okayama University Medical School, Okayama, 700-8558 Japan; 5 Kawasaki Medical School, Kurashiki, Okayama 701-0192 Japan; 6 Koneru Lakshmaiah Educational Foundation, Green Fields, Vaddeswaram, Andhra Pradesh 522302 India; 7 Stanford University, Stanford, CA 94305 USA

**Keywords:** Biochemistry, Cell biology, Molecular biology

## Abstract

We herein elucidate the function of SARS-CoV-2derived 5'UTR in the human cells. 5'UTR bound host cellular RNAs were immunoprecipitated by gRNA-dCas13 (targeting luciferase RNA fused to SARS-CoV-2 5'UTR) in HEK293T and A549 cells. The 5'UTR bound RNA extractions were predominantly enriched for regulating lipid metabolism. Overexpression of SARS-CoV-2 5'UTR RNA altered the expression of factors involved in the process of the human Mevalonate pathway. In addition, we found that HMG-CoA reductase inhibitors were shown to suppress SARS-CoV-2 5'UTR-mediated translation activities. In conclusion, we deduce the array of host RNAs interacting with SARS-CoV-2 5'UTR that drives SARS-CoV-2 translation and influences host metabolic pathways.

## Introduction

The outbreak of a novel strain of coronavirus—SARS-CoV-2 causing severe respiratory illness designated as COVID-19, surged as a large-scale global pandemic claiming more than 1 million lives while requiring intensive care hospitalizations for 2–10% of the infected cases worldwide^1^. Accumulated data corroborated since a year of the pandemic has shown COVID-19 patients to present diverse symptoms with severe immune dysregulation of unknown incentives^2–5^.

It is noteworthy that a recent meta-analysis establishes a positive link between COVID-19 vulnerability with obesity^6–10^. Hospital-based studies showed that people with obesity were 113% more likely to contract SARS-CoV-2 and 74% of them required intensive care admissions^11^. Furthermore, patients in the excessive BMI with metabolic-related fatty liver disease demonstrated an enhanced risk of severe COVID-19 disease^12^. These studies explicitly indicate obesity as a potential comorbid condition for COVID-19 affecting the overall course of the disease by influencing viral load, tissue damage, and mortality.

Although the rate of mutation in SARS-CoV-2 is slow (two single nucleotide substitutions per month)^13^, nevertheless, more than 12,000 mutations have been documented so far (www.gisaid.org). Among these, only a few of them result in the virus's protein variations which make it competent for human-to-human transmission and was responsible for severe illness, hospitalization and mortality rate especially associated with Beta and Delta variant^4,5^.

Viruses like SARS-CoV-2 exploit the complexity of eukaryotic initiation to gain access to the host machinery for protein production^14^. The genome of SARS-CoV-2 consists of approximately 30 kb of linear, non-segmented, 5′-capped, polycistronic, polyadenylated RNA molecules, comprising a total of 14 open reading frames (ORFs)^15^. SARS-CoV-2 genomic RNA is capped at the 5'end, and translation initiation efficiency is regulated by a stable hairpin structure near the cap structure^16,17^. The 5′ UTR of many RNA viruses has a unique stem-loop structure and is known to recruit important host translation regulatory factors including proteins and RNA, but studies on the physical binding of such factors to theSARS-CoV-2 RNA remains to be elucidated.　Understanding the interaction between viral RNA and host factors will be useful in seeking to develop effective therapeutic targets for this disease^18,19^.

In the present study, employing CRISPR/dCas13 aided targeting and immunoprecipitation of SARS-CoV-2 5'UTR bound host RNAs, we herein provide the possible model of regulation of SARS-CoV-2 in human cells and the way it affects the host cellular metabolism.

## Results

### Selection of high prevalent 5'UTR region of SARS-CoV-2

In order to understand the molecular influence of SARS-CoV-2 in human cells, we introduced the 5’UTR leader sequence of SARS-CoV-2 into the host cell (A549 and HEK293T cells). This region is important to facilitate the understanding of (1) the array of host RNAs that would interact with5’UTR leader in regulating translation of the viral genome and (2) the host cell types that are more susceptible for SARS-CoV-2 infection and replication. However, considering the fact that hypermutation in 5’UTR regions is typical to viral genomes it was essential to use only those 5’UTRs in the present experiment which have large geographic expansions i.e. prevalent across the diverse ethnicities and conserved since the first outbreak.

**Figure 1:**
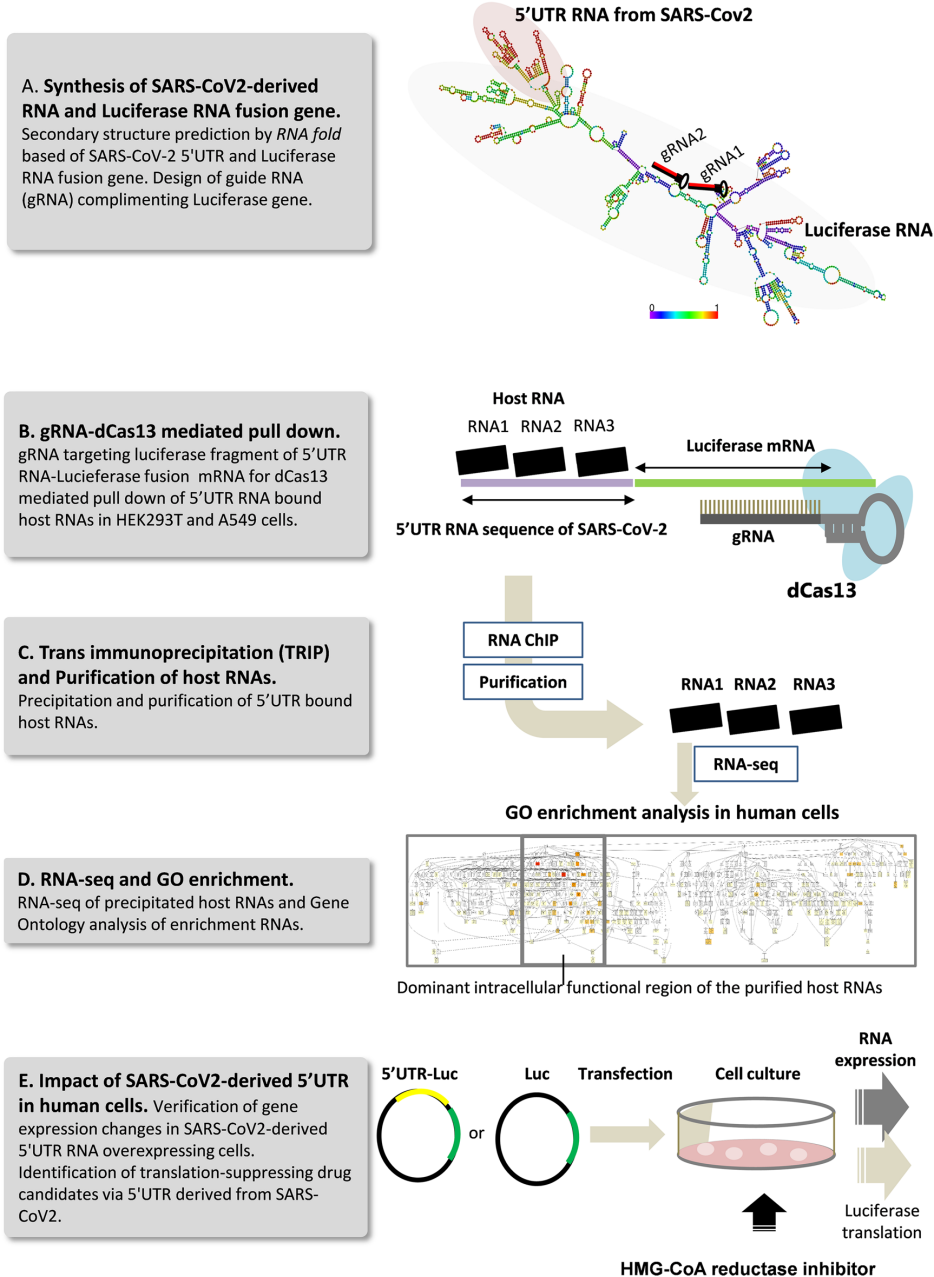
Workflow of the study.

Numerous reports have been made available on sequence variation and phylogenetics of SARS-CoV-2, and have been constantly updated on https://nextstrain.org/sars-cov-2/.The data snapshot containing a quality filtered subset of 3,434 available data points from the site is provided in Supplementary Fig. 1. Among the 264nt sequences in the 5'UTR region of SARS-CoV-2, the two derived from the 210 G > T and 241C > T point mutations were proven to be the most prevalent in almost all the infected cases worldwide. Interestingly, the 241 T mutant was also detected at the first outbreak in Wuhan China in 2019, while other 5'UTR mutations, although been reported, did not lead to clade formations. It also deviates from the binding segment of the virus-derived Nsp1 protein, presumably it benefits viral survival and replication in host cells. Given this literature, we hence selected 5'UTR sequence for our study and henceforth pursued to identify the cluster of host RNAs that interact with this 5’UTR that aids translation and regulation of SARS-CoV-2 genome in human cells. The complete workflow of this study is demonstrated in Fig. 1.

### Translation activation by 5'UTR RNA derived from SARS-CoV-2

**Figure2:**
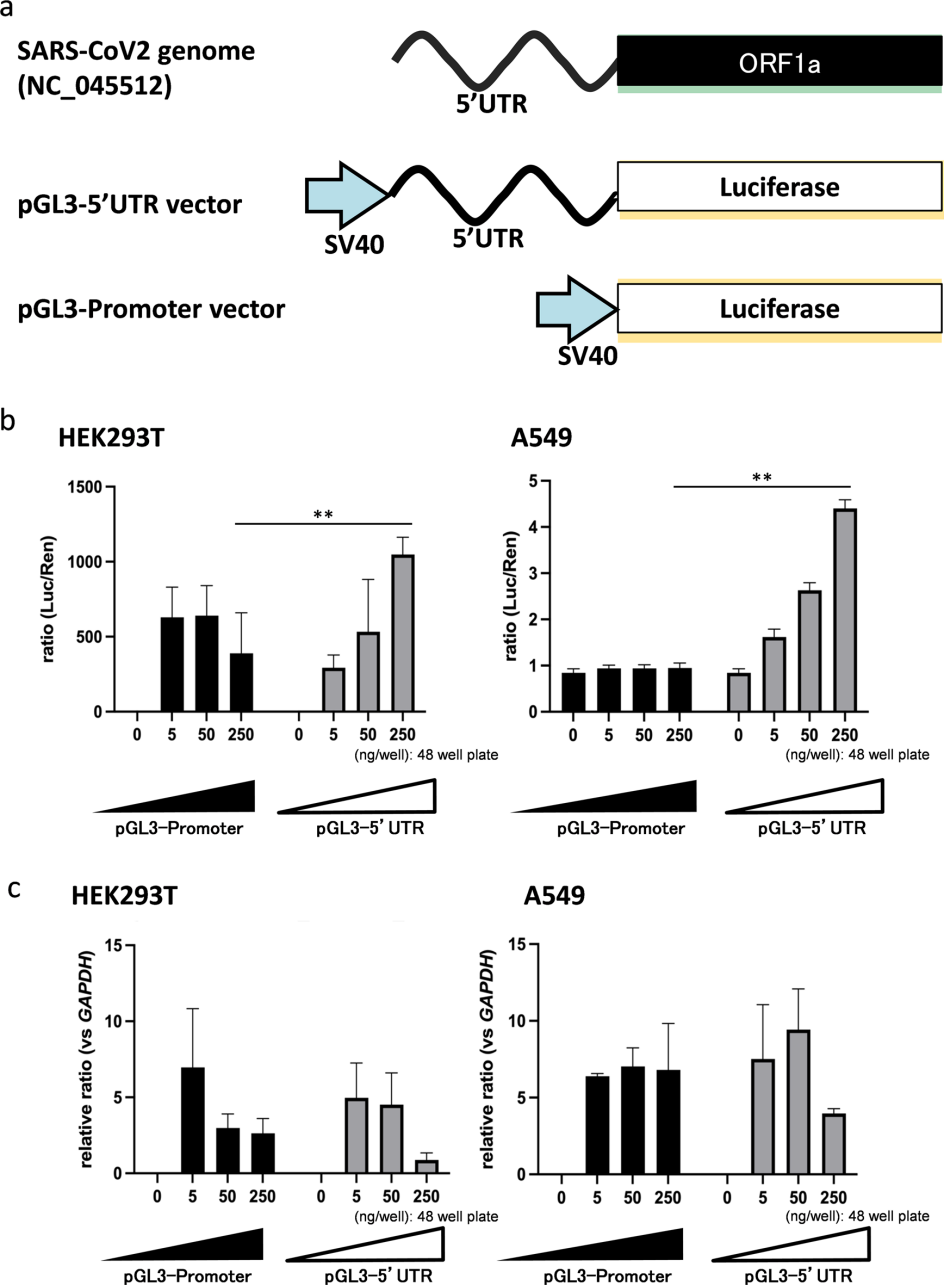
pGL3-5'UTR construct and Luciferase reporter assay. (**a**) Vector construction with SARS-CoV-2 5'UTR insert in pGL3 luciferase vector. The genomic architecture of SARS-CoV-2 starts with 5’ UTR followed by ORF1 and structural genes. To elucidate the translational mechanism in host cells, 5’ UTR segment of the SARS-CoV-2 was inserted in the pGL3 vector between SV40 promoter and luciferase gene. Luciferase gene is a reporter for the transfection efficiency and expression of 5’ UTR. Further, luciferase RNA forms the target for crRNA-dCAS13 facilitating TRIP for 5’UTR bound host translational RNAs. (**b**) Luciferase protein expression was measured as a luminescence activity of luciferase protein. Upon introducing the pGL3-5'UTR vector, relative luciferase activity increased more than ten times to the equivalent amount of pGL3-promoter. Note that the SARS-CoV-2derived 5'UTR synergistically activated translation in an intracellular factor-dependent manner. The relative luciferase activity was calculated by dividing the activity of Renilla reniformis luciferase as internal control. The mean and standard error of the three independent experiments is shown. The Mann–Whitney U test was used for statistical significance (p < 0.05). (**c**) The expression of luciferase RNA in both SARS-CoV-2 5'UTR and pGL3 luciferase vectors brought about by the SV40 promoter was measured by qPCR. Note that The luciferase transcript expressed from SARS-CoV-2 5'UTR and pGL3 luciferase vectors (in both A549 and HEK-293 T cells) does not quantitatively differ significantly, implying that the expression of luciferase transcript is independent of the inclusion of 5'UTR in the pGL3vector. The expression results were expressed as fold relative to the housekeeping internal control GAPDH gene expression. The degradation of the RNA progressed when 250 ng of plasmid was introduced.

The sequence of the 5'UTR region of SARS-CoV-2 (isolated fromthe Wuhan-Hu-1 genome (NC_045512)) was oligo-synthesized. The 5'UTR sequence of SARS-CoV-2 (Wuhan-Hu-1, complete genome, Sequence ID: NC_045512.2) consists of the following 264 nucleotides, including the Kozak sequence: “AUUAAAGGUUUAUACCUUCCCAGGUAACAAACCAACCAACUUUCGAUCUCUUGUAGAUCUGUUCUCUAAACGAACUUUAAAAUCUGUGUGGCUGUCACUCGGCUGCAUGCUUAGUGCACUCACGCAGUAUAAUUAAUAACUAAUUACUGUCGUUGACAGGACACGAGUAACUCGUCUAUCUUCUGCAGGCUGCUUACGGUUUCGUCCGUGUUGCAGCCGAUCAUCAGCACAUCUAGGUUUCGUCCGGGUGUGACCGAAAGGUAAGCCACC”. The synthesized sequence was cloned in Luciferase reporter pGL3-Promoter Vector (Promega: E1761) specifically between SV40 promoter sequence and luciferase gene sequence to express the 5'UTR-fused-luciferase RNA (Fig. 2a). This bonafide vector was transfected into human cell lines HEK293T and A549 with a concentration gradient and the expression was measured as a function of luminescence from the translation of luciferase protein. In the present study, HEK293T cells were used as reference cells for being easy to transfect with the pGL3 vector and suitable for overexpressing 5'UTR RNA of SARS-CoV-2. Interestingly, luciferase RNA fused to SARS-CoV-2-derived 5'UTR showed stronger luciferase activity in HEK293T cells and A549 cells in contrast to cells expressing luciferase RNA alone (Fig. 2b). Intriguingly, there is no discernible difference between the luciferase transcripts produced by the SARS-CoV-2 5'UTR and the pGL3 luciferase vectors (in both A549 and HEK-293 T cells), suggesting that the expression of the luciferase transcript is not dependent on the presence of the 5'UTR in the pGL3vector. This finding suggests that SARS-CoV-2 5'UTR is solely responsible to enhance translational efficiency by serving as a motif for binding of host translational factors to code viral proteins in the human cells (Fig. 2c). Further, neither the introduction of the pGL3-promoter vector nor the pGL3-5'UTR vector affected cell viability (Supplemental Fig. 2).

### gRNA-dCas13 assisted Trans RNA Immunoprecipitation (TRIP) of host regulatory RNA bound to 5’UTR of SARS-CoV-2

**Figure 3:**
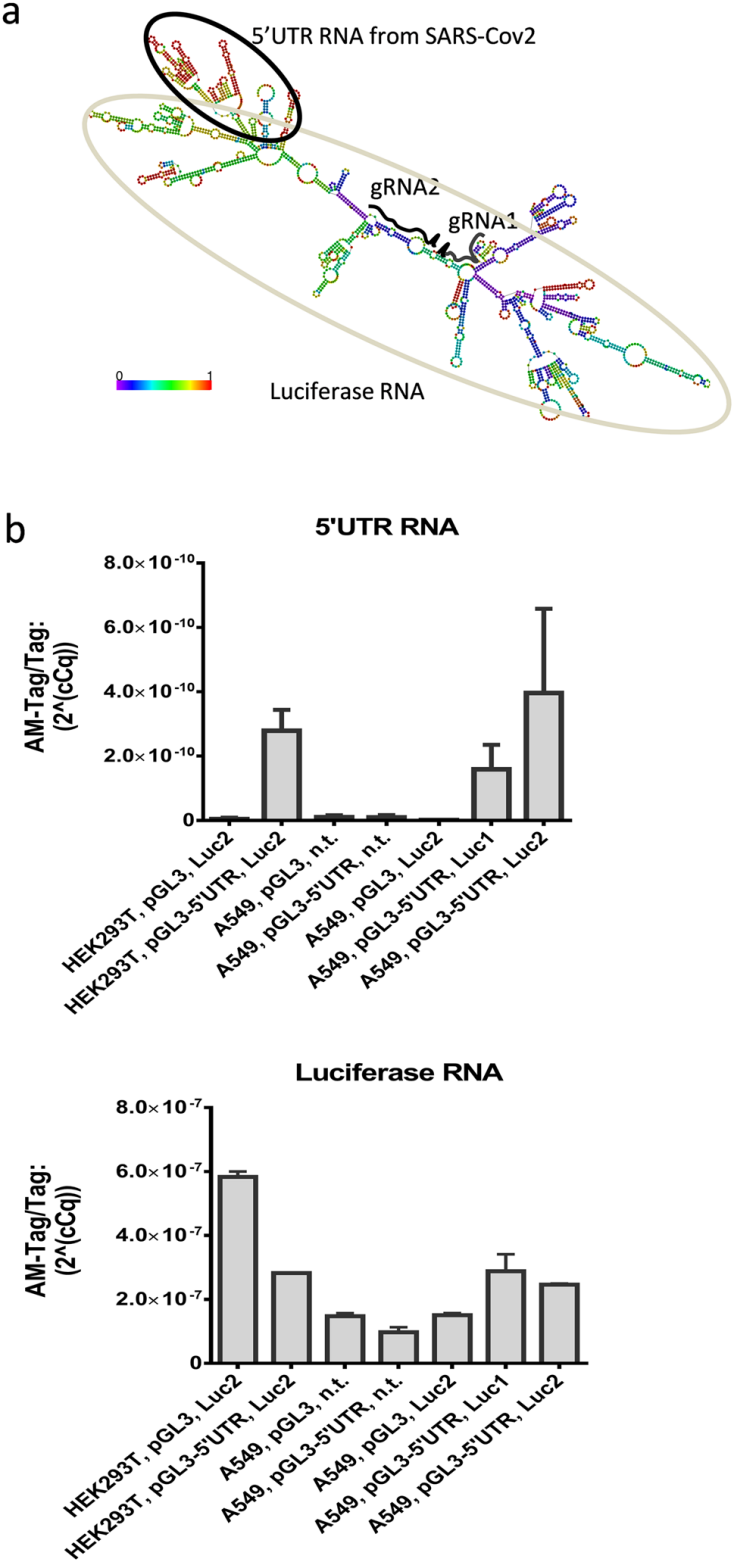
RNA-immunoprecipitation of 5-UTR bound host RNA. (**a**) Prediction of luciferase mRNA-5'UTR fusion. Two regions in the luciferase RNA were selected for target by 28 nt gRNAs–gRNA1 and gRNA2. (**b**) gRNA-dCas13 mediated RNA immunoprecipitation of host RNA bound to 5'UTR sequences. RT-qPCR was performed to determine the accumulation of immunoprecipitated 5'UTR sequence (Top) and luciferase RNA (bottom) via gRNA-dCas13 mediated targeting of luciferase RNA. The host RNA bound to 5’UTR was immunoprecipitated and the enrichment factor was calculated as 2 (− ΔΔCt [RIP/background]). The mean and standard error of the three independent experiments is shown. Statistical analysis was performed using a two-sided paired t-test. *Indicates a significant difference from the control sample at a value of p < 0.05. HEK293T cells (first and second white bars from the left) and A549 cells (third to seventh grey bars from the left).

Following our investigation which showed the significant roles of 5’UTR in enhancing translational efficiency, our subsequent pursuit was to determine the cluster of hostcellular RNAs that regulate SARS-CoV-2 genome by interacting to5’UTR sequence. To achieve this, we first predicted the structure of 5'UTR fused luciferase RNA and confirmed that 5'UTR RNA formed a secondary structure independent of luciferase RNA. Next, in order to pull down host RNAs bound to 5' UTR (by TRIP), we targeted luciferase fraction of 5'UTR-luceferase fused RNA by gRNA-dCas13. We designed two candidate single-strand regions for designing guide RNAs (gRNA) in the luciferase RNA. The gRNAs targeting these regions were named Luc1 and Luc2, respectively (Fig. 3a). The dCas13 gene sequence was inserted into the AAVS1 gene region of HEK293T cells and A549 cells by homologous recombination, followed by the selection of vector inserted cells with 10ug/ml puromycin for 24 h incubation. Next, lentivirus vectors expressing RFP and Luc1-gRNA or Luc2-gRNA were inserted in each cell, and the cells expressing RFP were collected by cell sorting. In the following experiment, we introduced pGL3-5'UTR vector or pGL3-promoter vector into cell lines expressing dCas13 and Luc1-gRNA or Luc2-gRNA, and performed RNA Immunoprecipitation (RIP) using AM-Tag antibody (pAb) (Active Motif: 61678). With qPCR we confirmed that the immunoprecipitated RNA libraries contained transcribed SARS-CoV-2-derived 5'UTR RNA and/or luciferase RNA. SARS-CoV-2derived 5'UTR RNA and luciferase RNAs were not detected in cells those expressing non-targeting gRNAs (gRNAs not target-specific for luciferase RNA) (Fig. 3b). Hence with gRNA-dCas13 assisted TRIP method we extracted 5' UTR bound host RNA clusters that are crucial in the translation of SARS-CoV-2 genome.

### 5'UTR bound host regulatory RNA predominantly contains genes for lipid metabolism

**Figure 4:**
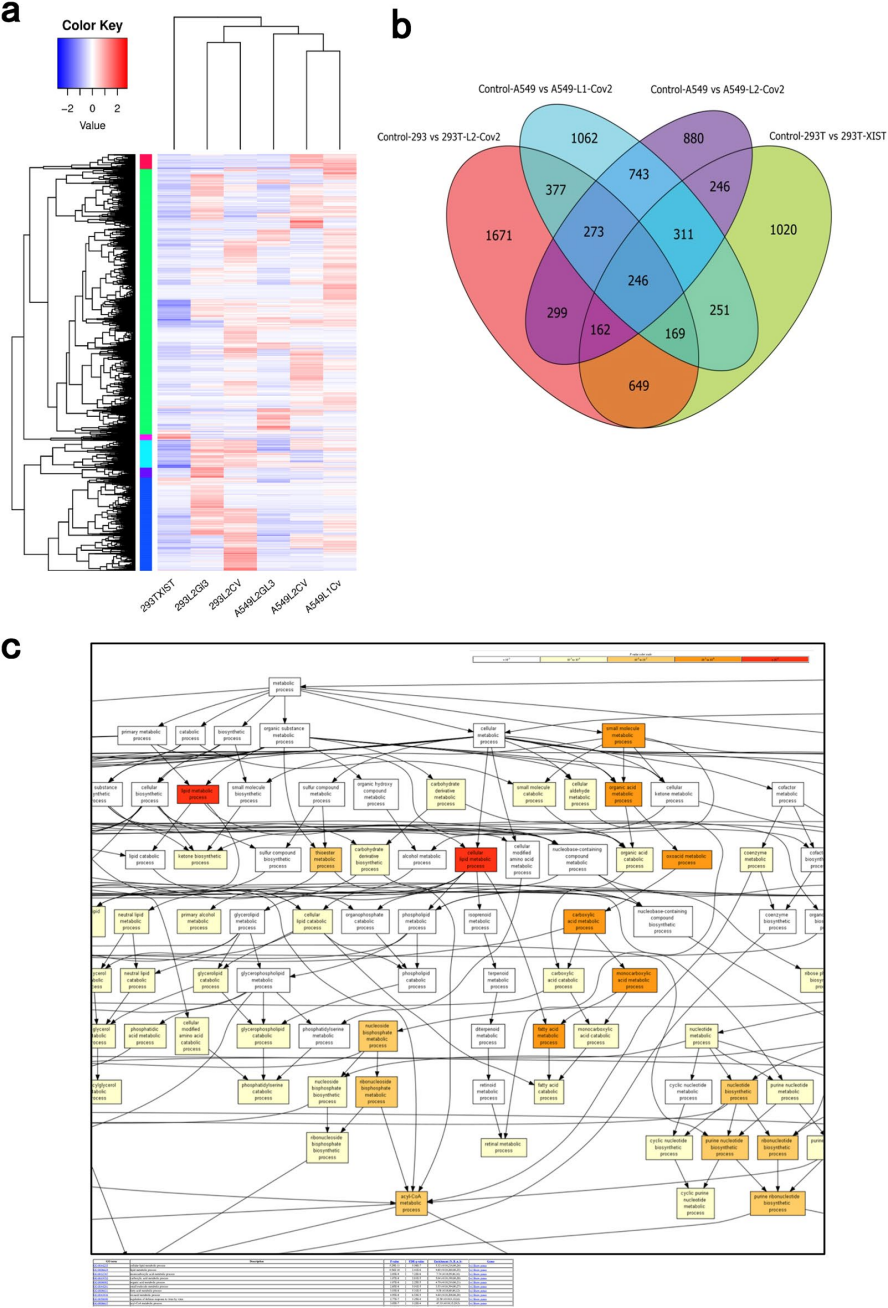
Differential expression by RNA seq analysis. (**a**) A set of four samples was analyzed for RNA-seq analysis. Each lane represents Luciferase RNA-immunoprecipitation sample having no 5'UTR sequence (293L2GL3 and A549L2GL3) as controls, and Luciferase-RNA fused with 5'UTR (293L2CV, A549L2CV, and A549L1Cv) as test samples. (L1 or L2 aregRNAs targeting Luciferase RNA). Heatmap analysis of differentially enriched genes shows enrichment in 293L2CV, A549L2CV, and A549L1CV immunoprecipitated samples. (**b**) Common and differentially expressed genes in test and control are shown in the Venn diagram. Set of unique genes in the test sample (293L2CV, A549L2CV, and A549L1Cv) were significantly enriched (p < 0.05) compared to the control (293L2GL3 or A549L2GL3). 293T-XIST is anRNA-immunoprecipitation sample which served as a negative control (HEK293T cells expressing lncRNA-XIST immunoprecipitated by gRNA-dCas13 pull down). The genes enriched in Control-293 vs. 293T-L2-CoV2, Control-A549vs. A549-L1-CoV2, and Control-A549-L2-CoV2 are human intracellular factors that 5'UTR of SARS-CoV-2. (**c**) Genes enriched in Control-293 vs 293T-L2-CoV2 dataset were ranked according to their differential enrichment and the resulting enriched GO terms were visualized using a DAG graphical representation (increase in enrichment degree is shown as a function of white to red gradient).

Host regulatory RNAs bound to 5'UTR extracted from TRIP were further subjected to ultra-low input RNA sequencing. In the cluster analysis of differentially enriched genes, we found that the RNA group that interact with SARS-CoV-2 5'UTR RNA in A549 cells belong to a different cluster for the RNA group that binds to Luciferase RNA (Fig. 4a). Similar enrichment differences between RNA group that binds to the 5'UTR RNA of SARS-CoV-2 and the control group that binds only to the RNA of luciferase could be confirmed in HEK293T cells as well (We confirmed the duplication of genes that are differentially enriched in each group compared to the control group or genes that are differentially enriched in multiple groups). We found that more than 1000 RNA genes that bind to the 5'UTR of SARS-CoV-2 are common to A549 cells and HEK293T cells (Fig. 4b). Further, we performed Gene Ontology enrichment analysis of RNA genes that bind predominantly (p-value < 0.01) to 5'UTR of SARS-CoV-2 extracted from HEK293T cells and A549. The top 5 GO terms identified in the analysis were (a) lipid metabolic process, (b) cellular lipid metabolic process, (c) small molecule metabolic process, (d) organic acid metabolic process and (e) oxoacid metabolic process (Fig. 4c and Supplement Table 1).

Next, we performed peak calling followed by motif analysis in order to find the sequence motifs in the 5'UTR which binds to host proteins (Supplement Fig. 3 and Supplementary Table 2). From the concentrated RNA motif, respectively 12 and 14 types of RNA-binding proteins were extracted from HEK293T and A549 cells. The proteins commonly observed in both cells were RBM38, BRUNOL4, BLUNOL5, and TARDBP. Although indirectly, these proteins have been shown to be involved in lipid metabolism and fat biosynthesis^20–24^. However to establish the specific role of these proteins in connection to 5’UTR of SARS-Cov2 RNA in regulating lipid metabolism still remains elusive and requires additional targeted metabolic investigations, nevertheless, this observation suggests that SARS-CoV-2 5'UTR may alter the lipid metabolic process in the host. Therefore from the given analysis it is suggestive that SARS-CoV-2 virus uses 5'UTR to interact with various protein and regulatory RNAs to sneak into the human intracellular environment. It may be thought of as "the takeover of RNA metabolism" mechanism in the human cell by the SARS-CoV-2 virus.

#### 5'UTR-mediated translation influences Mevalonate pathway

**Figure 5:**
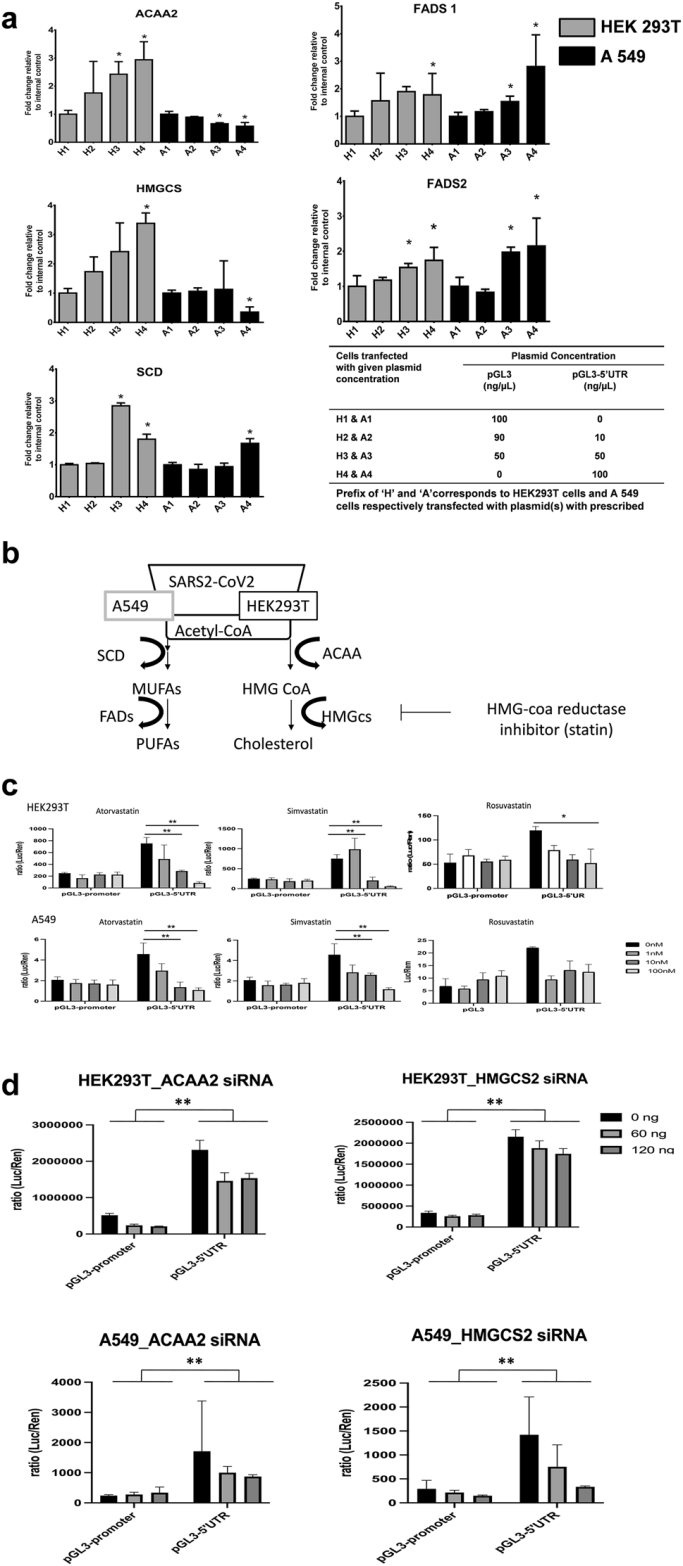
Overexpression of SARS-CoV-2–5'UTR of alters genes expression involved in the mevalonate pathway in HEK293Tand A549 cells. (**a**) 1 [micro]g of pGL3-promoter and pGL3-5'UTR vectors were transfected into cells and cultured in 6 well plates (cell density: 0.3 × 10^6^ cells/ml). Total RNA was extracted from the transfected cells and the expression of lipid metabolism genes was analyzed by qPCR. As shown, upon overexpression of 5'UTR, the expression of ACAA2and HMGCSis significantly altered (p < 0.05). Three independent trials were performed. Results are shown as mean ± SD, and Student's t-test was used as a test of significance. The expression results were expressed as fold relative to house-keeping internal control GAPDH gene expression. The y-axis is fold change which is the ratio of cells transfected with pGL3-promoter only to cells transfected with pGL3-5'UTR. (**b**) Pathways showing overexpression of 5'UTR of SARS-CoV-2 triggers Acetyl-CoA pathway, which can be repressed by HMG-CoA reductase inhibitors like statins. (**c**) 1 [micro]g of pGL3-promoter and pGL3-5'UTR vectors was introduced in cells cultured in 24 well plates. HMGCoA reductase inhibitors were added to the culture solution at a concentration range of 1 nM, 10 nM, 100 nM and 1000 nM. Following incubation for 48 h, the luciferase activity was measured. Y axis is relative luciferase activity which was calculated using the ratio of luciferase to Renilla reniformis luciferase as the internal control. (**d**) The luciferase activity was measured by transfecting 60 ng and 120 ng of esiRNA of HMGCS2 and ACAA2 into HEK293T and A549 cells, respectively. To assess the effect on the 5'UTR, the pGL3-promoter and luciferase activity from each adjacent well into which the pGL3-5'UTR vector was introduced were subtracted. The internal control used was the sum of each value. The results are shown as mean ± SD and tested for significance by Student's t-test. The internal control was the sum of the respective values. Y axis is relative luciferase activity which was calculated using the ratio of luciferase to Renilla reniformis luciferase as the internal control. Results are shown as mean ± SD, and Student's t-test examined significance. Statistical analysis data are in Supplement Information Table 3.

Following our previous investigation, which revealed that RNAs interacting with the 5'UTR are enriched in lipid metabolism, we investigated whether factors involved in lipid metabolism are altered when SARS-CoV-2-derived 5'UTR RNAs are abundantly expressed in human cells. Therefore, we introduced pGL3-5'UTR or pGL3-Promoter vectors into HEK293T cells and A549 cells, and quantified the RNAs. Surprisingly, significant changes in the mRNA expression level were observed for ACAA2, HMGCS, and FADS1/2 genes which are enriched in the Mevalonate and Polyunsaturated fatty acid biosynthesis pathway, and remarkably, HMGCS expression was highly upregulated at HEK293T cell (Fig. 5a). The Mevalonate pathway was predominant in HEK293T cells, whereas the Polyunsaturated fatty acid biosynthesis pathway was predominant in A549 cells (Fig. 5a). Since we had a set of enriched genes that bind to 5'UTR of SARS-CoV-2 in human cells, we further proceeded for drug repositioning by target identification. We used TargetMine program (https://targetmine.mizuguchilab.org) which aided the identification of suitable targets. As a result, four interacting compounds (Atorvastatin, Simvastatin, Rosuvastatin and Pravastatin) that can be expected to act (p-value < 0.05) on the enriched RNA were identified. Amongst all, statins (p-value < 0.01) have been identified as optimal and ready-to-use compounds to treat antiviral growth in COVID-19 patients (Fig. 5b). To investigate, whether suppression of translational activity by 5'UTR of SARS-CoV-2 can be introduced, we added over-the-counter drug HMG-CoA reductase inhibitors after overexpressing the pGL3-5'UTR or pGL3-Promoter vector into HEK293T cells and A549 cells. It was interesting to observe that 5'UTR SARS-CoV-2-mediated translational activity in both HEK293T cells and A549 cells were significantly suppressed upon treatment with 100 nM Atorvastatin (Fig. 5c). In addition in HEK293T cells, a translation inhibitory effect was observed upon treatment with 100nMRosuvastatin (Fig. 5c). Since both Atorvastatin and Rosuvastatin are hydrogen-bonded to Ser565 of HMG-CoA, it is thought that they have an auxiliary effect that other drugs do not have, which may be one of the reasons for their efficacies. Furthermore, to identify factors that affect the translational activity of luciferase proteins via the 5'UTR, we knocked down ACAA2 and HMGCS2 using MISSION® esiRNA (Sigma). Interestingly, knockdown of ACAA2 repressed the translational activity of luciferase proteins, whereas this was less pronounced for knockdown of HMGCS2 (Fig. 5d, Supplemental Fig. 4). Interestingly in close parallels to our study emphasizing relationship between the mevalonate pathway and viral infection, previous reports have shown that suppressing HMGCS2 or ACAA2 expression may suppress papillomavirus or poxvirus infection ^25,26^.

## Discussion

Since the outbreak of the COVID-19 pandemic in December 2019, its underlying molecular pathogenesis and clinical complications following the SARS-CoV-2 infection have been unceasingly investigated. Previous studies have shown that the COVID-19 patients suffer from excessive immune response termed “cytokine storm” with infiltration of cytokines, mononuclear cells, and monocytes^27,28^. However, in patients with bariatric diseases, the infiltration is extreme and prolonged that leads to acute tissue damage resulting in respiratory distress syndrome (ARDS)^7–9^. Although our in vitro studies were an artificial validation of the fusion of the 5'UTR with luciferase RNA, where in we found significant alteration in lipid metabolism in cells treated with 5'UTR of SARS-CoV-2. It remains obscure why lipid metabolism is altered upon SARS-CoV-2 infection making obese people more vulnerable to disease, however recent studies suggest preferential loss of lipids and amino acids associated enhanced infiltration of cytokines to be responsible for disease severity in COVID-19^29,30^. It is anticipated that further understanding of altered lipid metabolism in the virus-infected cells may help uncover the severity pattern in patients.

Recent structural and biochemical insights have shown 10- to 20-fold higher affinity of SARS-CoV-2 spike proteins to human ACE2 receptors than SARS-CoV^31^which possibly explains the higher transmissibility of COVID-19^32^. Therefore, an antibody-based therapeutic strategy that blocks the interaction site of SARS-CoV-2 and ACE2 receptor is anticipated to be effective^33^. Further, twophase-I trials (NCT04392219 and NCT04746183) have demonstrated that oral molnupiravir, aNovel Oral Anti-SARS-CoV-2 agent, is safe and well-tolerated at therapeutic doses. However, the latest analysis of a late- stage clinical trial of molnupiravirhas shown to reduce the risk of hospitalisation or death from serious illness by 30%^34^. Despite all curative interventions, Providing preliminary supportive care to the patients forms an effective disease management approach during the growing pandemic. Drug repositioning forms the rescue approach until a safe and reliable cure becomes available.

Elucidating the function of the 5'UTR region stands important as it forms the translational initiation region for host cellular factors to translate SARS-CoV-2 RNA. Although limited to translational activity from luciferase RNA fused to the 5'UTR of SARS-CoV-2, we observed significant changes in expression for genes related to mevalonate pathways and lipid metabolism. We herein show that HMG-CoA reductase inhibitors like Atorvastatin suppress the translational activation function of the 5'UTR region. Our cell-based investigation interestingly finds a good correlation with the previous clinical studies which showed that patients under the administration of HMG-CoA reductase inhibitors showed less aggravated symptoms for COVID-19^35–37^. We anticipate that short-term administration of HMG-CoA reductase inhibitors may likely suppress the exacerbation of SARS-CoV-2infected individuals. When the expression of ACAA2 was suppressed by siRNA instead of statin, it had a strong inhibitory effect on the translational activity of luciferase protein via 5'UTR in both cells. The proliferative capacity of HEK293T and A549 cells was also good in a statin or siRNA-treated environment with 5'UTR-mediated inhibition of translation of the luciferase protein (Supplementary Fig. 1). In terms of drug repositioning the use of statins in SARS-CoV-2 infected patients may be reasonable, however, the indication of drugs that inhibit ACAA2 expression may be more effective in repressing translation through the 5'UTR of SARS-CoV-2.

## Conclusion

In conclusion from the present study we herein elucidate the function of the SARS-CoV-2 5'UTR region in human cells which helps in understanding of the host transcriptome changes which may in turn help to predict the severity of the infection. Further elucidation of regulation of HMGCS2 or ACAA2 expressions including the results obtained from the present study may provide multiple antiviral drug development opportunities in the near future. We anticipate that the altered expression of genes as a result of infection may serve as a biomarker to predict the course of the disease. Our CRISPR/dCas13-based RNA genetic engineering approach as employed in the present study can also be extended to understand host cell response in other infectious diseases as well.

## Materials and methods

### Cell culture and transfection

HEK293T (ATCC CRL-3216) was a gift from M. Ladanyi (Memorial Sloan Kettering Cancer Center, New York) and A549 (ATCC CCL-185) cells was a gift from T. Takarada (Okayama University, Japan). These cells were grown in Dulbecco’s modified Eagle’s medium (Cellgro, D5796, Mediatech, Washington, DC) supplemented with 10% fetal calf serum and penicillin/streptomycin (Sigma). Plasmids were transfected into cells with GeneJammer (Agilent, 204130, Santa Clara, California, USA) according to the manufacturer’s protocol. All cell lines and the plasmid containing the SARS-CoV-2 viral genomic sequence were handled according to a protocol approved by the internal review committee and the ethics committee of Kawasaki Medical School and confirmed by the Minister of Education, Culture, Sports, Science and Technology of Japan (Accepted No.1020). We have not conducted experiments on any of the following which requires ethical clearances. (1) human embryos and gametes, (2) human embryonic stem cells and related materials, (3) clinical applications of stem cell, (4) Human recipients or donors of cells or tissue and (5) Animals experiment. The AAVS1_Puro_Tet3G_AM-dCas13 vector and eSpCas9(1.1)_No_FLAG_AAVS1_T2 plasmid (addgene:#79888) were transfected into HEK293T and A549 by ScreenFect A plus (https://screenfect.jp/screenfectaplus/) according to the manufacturer’s protocol. Tagging puromycin resistance to the open reading frame of AAVS1 together with the AM-dCas13 gene by homologous recombination maintains the physiological control of dCas13 gene expression^38^. pGL3-promoter or/and pGL3-5’UTR were lipofected into HEK293T or A549 by ScreenFect A plus according to the manufacturer’s protocol. The transfected cells were incubated for 48 h in the atmosphere of 5% CO_2_ at 37 °C. Finally, the infected cells were selected with 1 µg/mL puromycin (Thermofisher scientific).

### Plasmid preparation

Plasmids encoding human optimized Leptotrichia wadei Lwa dCas13a (WP_021746774.1) (full-length, following the am tag array with AM tag sequence: ATGTGCCAAGATCCTCAACGCAAAGGCAACGTGATACTCTCTCAGGCTTACGGGTGCCAAGATCCTCAACGCAAAGGCAACGTGATACTCTCTCAGGCTTAC) vector pENTR-dCas13 at GenScript (https://www.genscript.com/) and dCas13 sequence fused with AM-tag was subcloned into AAVS1_Puro_Tet3G_3xFLAG_Twin_step vector (addgene:Plasmid #92099) by gibson assembly protocol (Gibson Assembly Master Mix:NEB E2611S) to generate AAVS1_Puro_Tet3G_AM-dCas13 vector. The crRNA backbone vector (pLKO5.U6.crRNA.tRFP.v1) were synthesized at GenScript (https://www.genscript.com/). The two guide RNA sequences (Luc1 and Luc2) targeting luciferase were each subcloned into the crRNA backbone vector (pLKO5.U6.crRNA.tRFP.v1) after restriction enzyme treatment with BsmbI. Lentiviral particles were produced by transient transfection of HEK293T cells using the calcium-phosphate transfection method. Viral constructs were co-transfected with pMD2.G (Addgene plasmid 12259) and psPAX2 (Addgene plasmid 12260). Lentiviral particles were concentrated using ultra centrifugation. Nuclease dead Cas13a was designed to make base substitutions at R474A and R1046A. Nuclease dead Cas13a was designed to make base substitutions at R474A and R1046A. pGL3-5’UTR reporter plasmid containing 5’UTR sequence of the SARS-CoV-2 was also synthesized at GenScript and ligated into pGL3-promoter vector (Promega, E1761, Madison, Wisconsin, USA). Luc1-crRNA and Luc2-crRNA were constructed using BsmBI fragments sub-cloned into crRNA backbone vector pLKO5.U6.crRNA.tRFP.v1. The following sequence was inserted into the pGL3-5'UTR vector by cutting the sequence in the MCS of the pGL3-promoter vector with HindIII and NcoI restriction enzymes. ATTAAAGGTTTATACCTCCCAGGTACACACACCAACACACTTCTTCGATCTCTGTGTAGATCTGTCTCTTCAACGACTCTTAAATCTGAATCTGTGTGTCTCGCTGCATGCTTAGTGCACTCACGCAGTAT AATTAATACTAATACTGTCGTGTGACAGACGAGTAACTCGTCTATTCTCTGCAGGCTGTCTACGTCTTCGTCGTCGTCTCGTCGTCGTCGTCGTCGTCTTCGTCGTCGTCTTCGTCTTCGTCGTCGTCATCAGCACATCTAGGTTTCGTCC GGGTGTGACCGAAAGGTAAGCCACC". The Kozak sequence was the same as that from SARS-CoV-2 and the pGL3-promoter vector.

### esiRNA transfection

For transfection, all esiRNAs were brought to the same concentration by diluting in TE-buffer and were transfected by Lipofectamine™ RNAiMAX Transfection Reagent (Thermo Fisher: 13778030).

### Lentiviral transcription

To generate lentivirus vectors, constructs carrying crRNA backbone vector (pLKO5.U6.crRNA.tRFP.v1) were co-transfected with VSV-G-expressing envelope plasmid (pMD2G) (Addgene plasmid # 12259) and retroviral Gag, Pol, Rev, and Tat-expressing packaging vectors (psPAX2) (Addgene plasmid # 12260) into the HEK293T cells using ScreenFect A plus. Following four days of incubation, the HEK293T and A549 cell lines were treated with filtrated supernatant of lentivirus. Both psPAX2 and pMD2G were the gift from Didier Trono, EPFL Lausanne (https://www.epfl.ch/labs/tronolab/). The complete method for Lentiviral production was followed using the protocol suggested in lentiviral toolbox at Didier Trono Lab (https://www.epfl.ch/labs/tronolab/wp-content/uploads/2019/06/LV_production.pdf).

### Endoribonuclease-prepared siRNA (esiRNA) transfection

For specific targeting of human ACAA2 and HMGCS2 esiRNAs (MISSION esiRNA, Sigma Aldrich) were used. All the esiRNAs were brought to the equimolar concentration by diluting in TE-buffer and were transfected by Lipofectamine™ RNAiMAX Transfection Reagent (Thermo Fisher: 13778030).

### Trans RNA immunoprecipitation (TRIP)

Nuclear extracts from cell lines previously fixed with 2% paraformaldehyde were used for immunoprecipitation using 30 µl of anti-AM tag antibody (Active Motif, 61677, Carlsbad, California, USA). RNA immunoprecipitation was performed as described previously^25^ except that the AM-tag fused dCas13-RNA complexes were eluted from the Protein A Dynabeads (Thermo Fisher Scientific, 10001D, Waltham, Massachusetts, USA) and treated with 20 μg of proteinase K at 45 °C for 1 h, then de-crosslinked at 65 °C for 1 h. The RNA was isolated using TRI reagent (Ambion, AM9738, Austin, Texas, USA) according to the manufacturer's instructions. After treatment with Turbo DNase (Ambion, AM2238), the RNAs were subjected to RT-qPCR using primers to detect RNA sequence of Luciferase and 5’UTR of SARS-CoV-2. The RNAs were further subjected to Ultra Low Input RNA-seq (GeneWiz, https://www.genewiz.com). The DNase treatment of the extracts was performed at 25 °C for 30 min using 20 U of Turbo DNase (Ambion, AM2238).

### Quantitative polymerase chain reaction

HEK293T cells or A549 cells were transfected with pGL3-promoer and pGL3-5'UTR plasmids. The amount of pGL3-promoter was reduced according to the amount of pGL3-5'UTR in order to equalize the amount of plasmid introduced. Poly(A)’RNA and total RNA were extracted from HEK293T and A549 using the RNeasy minikit (Qiagen, 74104, Hilden, Germany). cDNA was prepared by reverse transcription of 500 ng of total RNA using the PrimeScrip RT Master Mix (Takara, RR036A,Kusatsu, Japan). The resulting cDNAs were amplified using the QuantiTect SYBR Green PCR kit (Qiagen, 204143). The RT-PCR reaction was performed on StepOne Real-Time PCR Systems (Applied Biosystems, Foster City, California, USA). Quantitation, and calling of real-time amplification values were performed on StepOne Software Version 2.2 (https://www.thermofisher.com/). Expression data was normalized to glyceraldehyde-3-phosphate dehydrogenase (GAPDH) expression to the corresponding sample. Primer sequences are included in the supplemental information (Table 4).

### Luciferase reporter assay

pGL3-promoter vector and pGL3-5’UTR vector were used as a reporter gene for investigating the 5’UTR dependent translation activity. Plasmids were transiently transfected into HEK293T and A549 cells. The cells were harvested for 48 h after transfection, followed by which luciferase activity was analyzed with Dual-Luciferase reporter assay system (Promega, E1910) using MiniLumat LB9506 (Berthold Technologies). Luciferase activity and reporter activities were normalized in reference to cotransfected Rous sarcoma virus (RSV)-Renillareniformis luciferase expression plasmid as described previously^39^. The SD values were obtained from three trials of each test.

### Drug-induced translation suppression

For translation suppression effect induced by HMG-CoA reductase inhibitors, cells were maintained in the presence or absence of Atorvastatin Calcium, Simvastatin, Rosuvastatin Calcium Salt, Pravastatin Sodium Salt (FUJIFILM Wako Chemicals, 104451679902-63-9, 187-03361, 162-19821, Osaka, Japan) diluted in DMSO (1 nM–1000 nM) for 48 h.

### RNA seq and data analysis

Total RNA was sequenced on illuminaNovaSeq 6000 sequencing platform on a 2 × 150 bp flow cell. Reads generated from sequencing were saved in Illumina specific (Binary Base Call (BCL) file format. All the BCL files were converted to fastq files with Bcl2fastq v2.17.1.14 (https://sapac.support.illumina.com/downloads/bcl2fastq-conversion-software-v2-20.html) compatible for downstream sequence analysis programs. Prior to data analysis, the reads were filtered by eliminating low-quality reads (Phred score < 30) by FastQC (http://www.bioinformatics.babraham.ac.uk/projects/fastqc/), trimming reads those less than 75 bp by Trinnomatic v 0.36 (http://www.usadellab.org/cms/index.php?page=trimmomatic)^40^. Cutting adapter sequences by Cutadapt version 1.9.1 (https://cutadapt.readthedocs.io/en/v1.9.1/)^41^. The filtered reads were subsequently aligned to the Human reference GRCh38.p12 using Hisat2 v2.0.1 ^42^, followed by assigning the reads onto genomic features—exons, introns and intergenic regions. The mapped reads were visualized by Integrative Genomics Viewer Version 2.5.2 (https://software.broadinstitute.org/software/igv/)^43^. For assembly and predict alternative splicing StringTie v1.3.3b (https://ccb.jhu.edu/software/stringtie/)^44^ was used and ASprofile V1.0.4 (https://ccb.jhu.edu/software/ASprofile/)^45^ was employed for classification and quantification of the spliced reads. For de novo transcript assembly StringTie v1.3.3b was employed using the alignment bam files, the result of which was then subjected to comparison with existing annotation reference (gtf file) using Cuffcompare V2.2.1 (https://www.genepattern.org/modules/docs/Cufflinks.cuffcompare/7)^46^. Gene expression calculation was performed as a function of FPKM with a threshold of 0.1–1 based on read counts from HT-seq v 0.6.1(https://bioweb.pasteur.fr/packages/pack@HTSeq@0.6.1)^47^. Differential gene analysis was performed using the Bioconductor package DESeq2 V1.6.3 and edgeR V3.4.6 (https://bioconductor.org/packages/release/bioc/html/edgeR.html)^48^. The results from EdgeR analysis were further analyzed to determine genes with significant differential expression according to the criteria of fold change greater than 2 and qvalue (fdr, padj) less than 0.05. The GO functional enrichment analysis was performed by GOSeq 1.42.0 (https://bioconductor.org/packages/release/bioc/html/goseq.html)^49^. TopGO (https://bioconductor.org/packages/release/bioc/html/topGO.html)^50^ was applied for generating Directed Acyclic Graph (DAG) for results of enrichment analysis of the differentially expressed genes. DEXSeq (V1.18.4) (https://www.rdocumentation.org/packages/DEXSeq/versions/1.18.4) was used to assess differential expression of exons and alternative exon usage in alternative splicing^51^. Enriched gene list is included in the supplemental information.

## Supplementary Information

Supplementary Information 1.

Supplementary Information 2.

Supplementary Information 3.

Supplementary Information 4.

Supplementary Information 5.
