# Supplementary material for: An RNA-immunoprecipitation via CRISPR/dCas13 reveals an interaction between the SARS-CoV-2 5'UTR RNA and the process of human lipid metabolism
Source: Sci Rep. 2023 Jun 27;13:10413. doi: 10.1038/s41598-023-36680-6 (PMC10300121; doi:10.1038/s41598-023-36680-6)
Supplement: Supplementary file 1 — Supplementary Information 1. [file 41598_2023_36680_MOESM1_ESM.docx]

**Supplementary Information**

**An RNA-immunoprecipitation via CRISPR/dCas13 reveals an interaction between the SARS-CoV-2 5'UTR RNA and the process of human lipid metabolism.**

Yurika Shimizu^1,2^, Srinivas Bandaru^1,6^, Mari Hara^1^, Sonny Young^3^, Toshikazu Sano^3^, Kaya Usami^4^, Yuta Kurano^5^, Suni Lee^1^, Naoko Kumagai-Takei^1^, Shogo Takashiba^2^,Shunji Sano^3^, Tatsuo Ito^1*^

1. Department of Hygiene, Kawasaki Medical School, 577 Matsushima, Kurashiki, Okayama, 701-0192, Japan.

2. Department of Pathophysiology - Periodontal Science, Okayama University Graduate School of Medicine, Dentistry and Pharmaceutical Sciences, Okayama, Okayama, 700-8525, Japan.

3. Department of Surgery, Division of Pediatric Cardiothoracic Surgery, University of California San Francisco, San Francisco, CA, USA.

4. Okayama University Medical School, Okayama, 700-8558, Japan.

5. Kawasaki Medical School, Kurashiki, Okayama, 701-0192, Japan.

6. Koneru Lakshmaiah Educational Foundation, Green Fields, Vaddeswaram, Andhra Pradesh 522302, India.

*Corresponding author

Tatsuo Ito

Department of Hygiene,

Kawasaki Medical University, 577 Matsushima, Kurashiki, Okayama, 701-0192, Japan.

[tataito@med.kawasaki-m.ac.jp](mailto:tataito@med.kawasaki-m.ac.jp).

**Figures**

**a**

**b**

**Supplementary figure 1.** (a) Diversity of SARS CoV-2 RNA genome sampled between December 2019 and November 2021 (Number of genomes sampled= 3434). (b) Diversity within SARS CoV2 5’UTR.

**Supplementary figure2.** **Cell viability assay**: Each cell line was plated on 96-well plates (1X10^3^ cells/well). According to the manufacturer's instructions, the Alamar Blue Cell Viability Assay Kit (Life Technologies) is used 48 hours after drug exposure or esiRNA introduction.

a

**Supplementary figure 3.** **Motif analysis of 5’UTR.** Shown here the motifs in the 5’UTR which binds to host proteins in HEK293T cells (top) and A549 cells (bottom).

**Supplementary figure 4.** **Knockdown of ACAA2 and HMGCS2 using MISSION® esiRNA (Sigma).** siRNA knockdowns the target transcripts, however RNAs those involved in lipid metabolism are not significantly affected by esiRNA silencing.


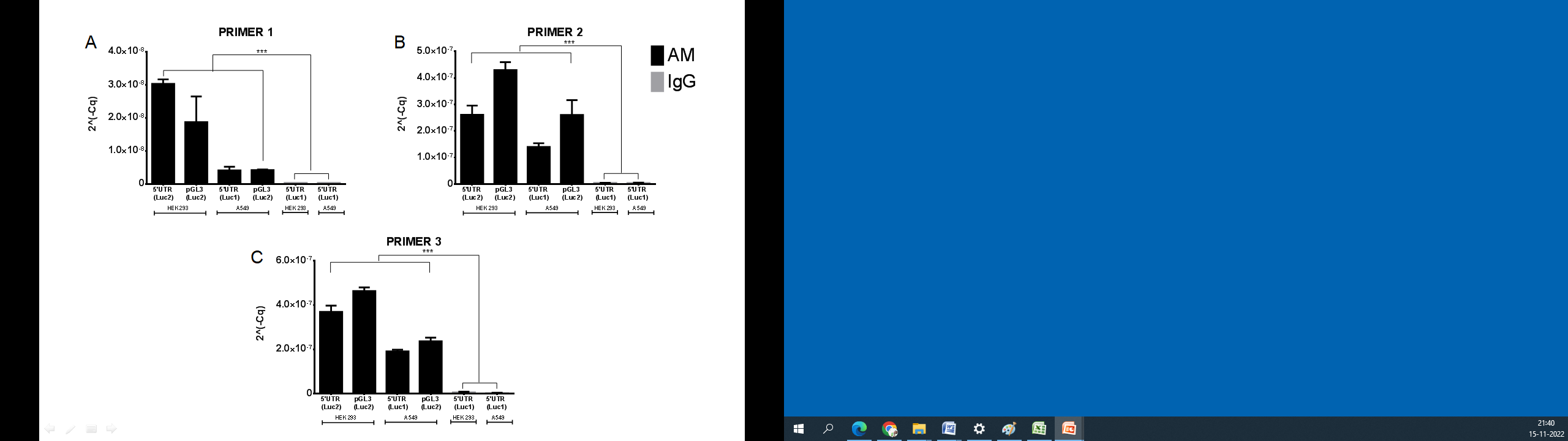


**Supplementary figure 5. Selection of primers for immunopreciptated RNA**. qPCR performed to determine luciferase sequences in immunoprecipitated RNA with three different primers (of all three, Primer 2 shows efficient detections of immunopreciptated RNAs). The Luciferase RNA bound to dCas13 (covalently tagged to AM Tag (Active Motif: 61678)) were detected significantly with selected primers than the IgG precipitated RNA (non-targeting control).

**Tables**

**Supplementary table 1.** Top 10 Gene ontology terms enriched for interacting 5’UTR of SARS-CoV-2.

| **GO term** | **Description** | **P-value** | **FDR q-value** |
| --- | --- | --- | --- |
| GO:0044255 | Cellular lipid metabolic process | 5.29E-11 | 5.58E-07 |
| GO:0006629 | Lipid metabolic process | 4.56E-10 | 2.41E-06 |
| GO:0032787 | Monocarboxylic acid metabolic process | 2.05E-09 | 7.22E-06 |
| GO:0019752 | Carboxylic acid metabolic process | 1.07E-08 | 2.81E-05 |
| GO:0006082 | Organic acid metabolic process | 1.07E-08 | 2.25E-05 |
| GO:0044281 | Small molecule metabolic process | 2.85E-08 | 5.01E-05 |
| GO:0006631 | Fatty acid metabolic process | 3.53E-08 | 5.31E-05 |
| GO:0043436 | Oxoacid metabolic process | 4.95E-08 | 6.53E-05 |
| GO:0050690 | Regulation of defense response to virus by virus | 2.77E-07 | 3.25E-04 |
| GO:0006637 | Acyl-CoA metabolic process | 3.05E-07 | 3.22E-04 |

**Supplementary table 2.** Host factors binding via consensus recognition motif to 5’ UTR of SARS- CoV-2.

| **Host Proteins** | **Recognition Motif in 5’ UTR** | **K-mer** | **Z-score**  **P-value (HEK293T)** | **Z-score P-value (A549)** |
| --- | --- | --- | --- | --- |
| RBM38 | gggugug | 4.321 | 7.77E-06 | 8.43E-08 |
| BRUNOL5 | uguguau | 3.912 | 4.58E-05 | 6.64E-08 |
| BRUNOL4 | uguguau | 3.838 | 6.20E-05 | 9.77E-06 |
| TARDBP | ugugug | 3.826 | 6.51E-05 | 1.38E-06 |

**Supplemental table 3.** siRNA induced silencing of ACAA2 and HMGCS2 in A549 and HEK293T cells

|  | **% of total variation ^a^** | **P value** | **Mean of pGL3-promoter** | **Mean of pGL3-5'UTR** | **SE of difference** | **95% CI of difference** |
| --- | --- | --- | --- | --- | --- | --- |
| **A549-siACAA2** | 34.83 | 0.0163 | 285.8 | 1195 | 325.7 | -1619 to -200.1 |
| **A549-siHMGCS2** | 32.46 | 0.005 | 219 | 836.2 | 179.1 | -1007 to -227.0 |
| **HEK293T-siACAA2** | 84.05 | <0.0001 | 322246 | 1769865 | 72549 | -1605689 to -1289549 |
| **HEK293T-siHMGCS2** | 96.58 | <0.0001 | 292305 | 1927797 | 53710 | -1752515 to -1518468 |
| **Two wayAnova, Alpha= 0.05, pGL3-promoter *vs.* pGL3-5'UTR.** | | | | | | |
| **^a^ Ratio of Luc/Ren for for all the concentrations (0 ng,60ng,120 ng) compared as group between pGL3-promoter and pGL3-5'UTR transfected cells.** | | | | | | |

**Supplementary table 4.** The primers used for amplification of transcript quantification of each gene

| **Transcript** | **Forward primer** | **Reverse primer** |
| --- | --- | --- |
| Luciferase_primer1 | 5' -GCTGGTGCCAACCCTATTCT -3' | 5' -GTGTTCGTCTTCGTCCCAGT -3' |
| Luciferase_primer2 | 5' -CGCATGCCAGAGATCCTATT -3' | 5 '-AGACGACTCGAAATCCACATATC -3' |
| Luciferase_primer3 | 5' -GTCCTATGATTATGTCCGGTTATGT -3' | 5' -GTCTTCGTCCCAGTAAGCTATG -3' |
| SARS-COV-2_5'UTR_1 | 5'-GACCGAAAGGTAAGCCACCA-3’ | 5'-TAGGCTGCGAAATGCCCATA-3’ |
| SARS-COV-2_5'UTR_2 | 5'-CCCAGGTAACAAACCAACCAAC-3’ | 5'-GACGAAACCGTAAGCAGCCT-3’ |
| *ACAA2* | 5' -GGGCACTGAAGAAAGCAGGA -3' | 5' -CGTGAACCAGGTGTGCAGTA -3' |
| *HMGCS2* | 5' -TGGTCTGTGGAGACATTGCC -3' | 5' -TATTGGGTACTCCGAGGCCA -3' |
| *ELOVL* | 5' -TGCTCTTCGAACTGGTGCTT -3' | 5' -ACCAGTGCAGGAAGATCAGC -3' |
| *FADS1* | 5' -GCGCCAGCAAATCCACTCC -3' | 5' -TCGTCCCAGGTGAAGTAGCG -3' |
| *FADS2* | 5' -GAAGCATAACCTGCGCACC -3' | 5' -ACCAATCAGCAGGGGTTTCA -3' |
| *SCD* | 5' -ACGCTTGTGCCCTGGTATTT -3' | 5' -CGATATCCGAAGAGGTGGGC -3' |
| *GAPDH* | 5' -GAGTCAACGGATTTGGTCGT -3' | 5' -TTGATTTTGGAGGGATCTCG -3' |
