# Supplementary material for: An RNA-immunoprecipitation via CRISPR/dCas13 reveals an interaction between the SARS-CoV-2 5'UTR RNA and the process of human lipid metabolism
Source: Sci Rep. 2023 Jun 27;13:10413. doi: 10.1038/s41598-023-36680-6 (PMC10300121; doi:10.1038/s41598-023-36680-6)
Supplement: Supplementary file 3 — Supplementary Information 3. [file 41598_2023_36680_MOESM3_ESM.pdf]

A

Name	gRNA sequence
Control(non target)	5'-CGGGAGACGTGACCGTCTCCTTTTTTTT-3′
Guide1 (Luc1)	5'-GCTGCGTTCTTTTTAGTCTCTCTAGGAG-3′
Guide2(Luc2)	5'-CCGGTTCTTCCCGCCTTTCTAGCGGCAC-3′

B

Gene Symbol	Sense primer (5' to 3')	Antisense primer (5' to 3')	NCBI Refseq	Species
**FADS1**	5′-GCGCCAGCAAATCCACTCC-3′	5'-TCGTCCCAGGTGAAGTAGCG-3′	NM_013402.7	Human
**FADS2**	5′-GAAGCATAACCTGCGCACC-3′	5'-ACCAATCAGCAGGGGTTTCA-3′	NM_001281501.1	Human
**ELOVL5**	5′-TGCTCTTCGAACTGGTGCTT-3′	5'-ACCAGTGCAGGAAGATCAGC-3′	NM_001242828.2	Human
**SCD**	5′-ACGCTTGTGCCCTGGTATTT-3′	5'-CGATATCCGAAGAGGTGGGC-3′	NM_005063.5	Human
**ACAA2**	5′-GGGCACTGAAGAAAGCAGGA-3′	5'-CGTGAACCAGGTGTGCAGTA-3′	NM_006111.3	Human
**HMGCS**	5′-TGGTCTGTGGAGACATTGCC-3′	5'-TATTGGGTACTCCGAGGCCA-3′	NM_001166107.1	Human
**GAPDH**	5′-GAGTCAACGGATTTGGTCGT-3′	5'-TTGATTTTGGAGGGATCTCG-3′	NM_001166107.1	Human
