# Supplementary material for: An RNA-immunoprecipitation via CRISPR/dCas13 reveals an interaction between the SARS-CoV-2 5'UTR RNA and the process of human lipid metabolism
Source: Sci Rep. 2023 Jun 27;13:10413. doi: 10.1038/s41598-023-36680-6 (PMC10300121; doi:10.1038/s41598-023-36680-6)
Supplement: Supplementary file 4 — Supplementary Information 4. [file 41598_2023_36680_MOESM4_ESM.pdf]

Table 1. Top 10 Gene ontology terms enriched for binding 5'UTR of SARS CoV2.

GO term	Description	P-value	FDR q-value
GO:0044255	Cellular lipid metabolic process	5.29E-11	5.58E-07
GO:0006629	Lipid metabolic process	4.56E-10	2.41E-06
GO:0032787	Monocarboxylic acid metabolic process	2.05E-09	7.22E-06
GO:0019752	Carboxylic acid metabolic process	1.07E-08	2.81E-05
GO:0006082	Organic acid metabolic process	1.07E-08	2.25E-05
GO:0044281	Small molecule metabolic process	2.85E-08	5.01E-05
GO:0006631	Fatty acid metabolic process	3.53E-08	5.31E-05
GO:0043436	Oxoacid metabolic process	4.95E-08	6.53E-05
GO:0050690	Regulation of defense response to virus by virus	2.77E-07	3.25E-04
GO:0006637	Acyl-CoA metabolic process	3.05E-07	3.22E-04

Table 2. Host factors binding via consensus recognition motif to 5' UTR of SARS CoV2.

Host Proteins	Recognition Motif in 5' UTR	K-mer	Z-score P-value (HEK293T)	Z-score P-value (A549)
RBM38	gggugug	4.321	7.77E-06	8.43E-08
BRUNOL5	uguguau	3.912	4.58E-05	6.64E-08
BRUNOL4	uguguau	3.838	6.20E-05	9.77E-06
TARDBP	ugugug	3.826	6.51E-05	1.38E-06
