# Supplementary material for: An RNA-immunoprecipitation via CRISPR/dCas13 reveals an interaction between the SARS-CoV-2 5'UTR RNA and the process of human lipid metabolism
Source: Sci Rep. 2023 Jun 27;13:10413. doi: 10.1038/s41598-023-36680-6 (PMC10300121; doi:10.1038/s41598-023-36680-6)
Supplement: Supplementary file 2 — Supplementary Information 2. [file 41598_2023_36680_MOESM2_ESM.pdf]

P-value color scale

> 10^{-3}	10^{-3} to 10^{-5}	10^{-5} to 10^{-7}	10^{-7} to 10^{-9}	< 10^{-9}

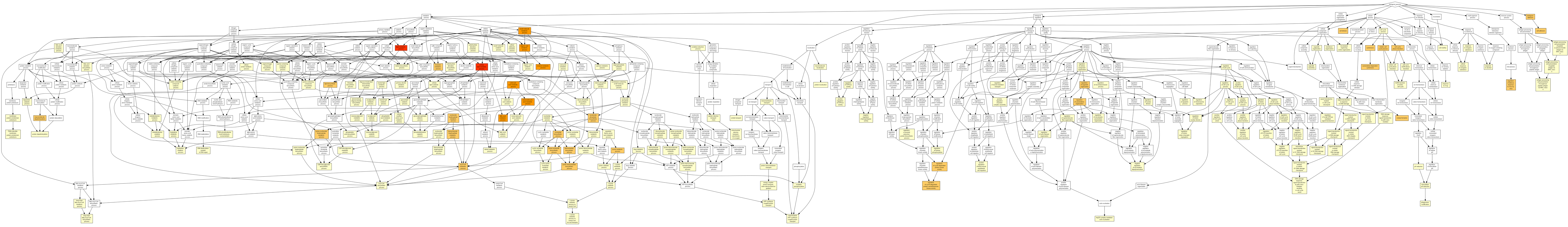

GO term	Description	P-value	FDR q-value	Enrichment (N, B, n, b)	Genes
GO:0044255	cellular lipid metabolic process	5.29E-11	5.58E-7	5.32 (4118,216,86,24)	[+] Show genes
GO:0006629	lipid metabolic process	4.56E-10	2.41E-6	4.60 (4118,260,86,25)	[+] Show genes
GO:0032787	monocarboxylic acid metabolic process	2.05E-9	7.22E-6	7.74 (4118,99,86,16)	[+] Show genes
GO:0019752	carboxylic acid metabolic process	1.07E-8	2.81E-5	5.04 (4118,190,86,20)	[+] Show genes
GO:0006082	organic acid metabolic process	1.07E-8	2.25E-5	4.79 (4118,210,86,21)	[+] Show genes
GO:0044281	small molecule metabolic process	2.85E-8	5.01E-5	3.53 (4118,394,80,27)	[+] Show genes
GO:0006631	fatty acid metabolic process	3.53E-8	5.31E-5	9.58 (4118,60,86,12)	[+] Show genes
GO:0043436	oxoacid metabolic process	4.95E-8	6.53E-5	4.60 (4118,208,86,20)	[+] Show genes
GO:0050690	regulation of defense response to virus by virus	2.77E-7	3.25E-4	22.50 (4118,9,122,6)	[+] Show genes
GO:0006637	acyl-CoA metabolic process	3.05E-7	3.22E-4	47.33 (4118,15,29,5)	[+] Show genes
GO:0035383	thioester metabolic process	3.05E-7	2.93E-4	47.33 (4118,15,29,5)	[+] Show genes
GO:0051301	cell division	5.79E-7	5.1E-4	2.56 (4118,131,454,37)	[+] Show genes
GO:1903047	mitotic cell cycle process	8.68E-7	7.05E-4	2.06 (4118,208,509,53)	[+] Show genes
GO:0000079	regulation of cyclin-dependent protein serine/threonine kinase activity	1.63E-6	1.23E-3	4.76 (4118,27,449,14)	[+] Show genes
GO:0034032	purine nucleoside bisphosphate metabolic process	1.8E-6	1.26E-3	35.50 (4118,20,29,5)	[+] Show genes
GO:0033865	nucleoside bisphosphate metabolic process	1.8E-6	1.19E-3	35.50 (4118,20,29,5)	[+] Show genes
GO:0033875	ribonucleoside bisphosphate metabolic process	1.8E-6	1.12E-3	35.50 (4118,20,29,5)	[+] Show genes
GO:0033043	regulation of organelle organization	1.87E-6	1.1E-3	1.79 (4118,403,429,75)	[+] Show genes
GO:0008610	lipid biosynthetic process	2.44E-6	1.36E-3	5.56 (4118,133,78,14)	[+] Show genes
GO:1904029	regulation of cyclin-dependent protein kinase activity	2.92E-6	1.54E-3	5.10 (4118,28,375,13)	[+] Show genes
GO:0007155	cell adhesion	3.13E-6	1.57E-3	1.89 (4118,161,800,59)	[+] Show genes
GO:0051493	regulation of cytoskeleton organization	3.26E-6	1.56E-3	2.33 (4118,165,429,40)	[+] Show genes
GO:0009152	purine ribonucleotide biosynthetic process	3.51E-6	1.61E-3	6.73 (4118,36,187,11)	[+] Show genes
GO:0022610	biological adhesion	3.96E-6	1.74E-3	1.87 (4118,162,800,59)	[+] Show genes
GO:1901293	nucleoside phosphate biosynthetic process	4.31E-6	1.82E-3	5.14 (4118,60,187,14)	[+] Show genes
GO:0009165	nucleotide biosynthetic process	4.31E-6	1.75E-3	5.14 (4118,60,187,14)	[+] Show genes
GO:0000910	cytokinesis	4.32E-6	1.69E-3	137.27 (4118,30,3,3)	[+] Show genes
GO:0061640	cytoskeleton-dependent cytokinesis	4.32E-6	1.63E-3	137.27 (4118,30,3,3)	[+] Show genes
GO:0044770	cell cycle phase transition	4.54E-6	1.65E-3	2.39 (4118,88,666,34)	[+] Show genes
GO:0006553	lysine metabolic process	4.95E-6	1.74E-3	588.29 (4118,2,7,2)	[+] Show genes
GO:0006164	purine nucleotide biosynthetic process	6.44E-6	2.19E-3	6.37 (4118,38,187,11)	[+] Show genes
GO:0042407	cristae formation	6.6E-6	2.18E-3	7.47 (4118,11,401,8)	[+] Show genes
GO:0009260	ribonucleotide biosynthetic process	8.7E-6	2.78E-3	6.21 (4118,39,187,11)	[+] Show genes
GO:0098734	macromolecule depalmitoylation	9.97E-6	3.09E-3	98.05 (4118,6,21,3)	[+] Show genes
GO:0016054	organic acid catabolic process	1.27E-5	3.82E-3	8.27 (4118,56,80,9)	[+] Show genes
GO:0046395	carboxylic acid catabolic process	1.27E-5	3.72E-3	8.27 (4118,56,80,9)	[+] Show genes
GO:0072521	purine-containing compound metabolic process	1.36E-5	3.88E-3	4.16 (4118,83,191,16)	[+] Show genes
GO:1901137	carbohydrate derivative biosynthetic process	1.37E-5	3.8E-3	3.75 (4118,96,206,18)	[+] Show genes
GO:0009150	purine ribonucleotide metabolic process	1.46E-5	3.95E-3	4.37 (4118,74,191,15)	[+] Show genes
GO:0006490	oligosaccharide-lipid intermediate biosynthetic process	1.52E-5	4.01E-3	49.61 (4118,3,83,3)	[+] Show genes
GO:0006488	dolichol-linked oligosaccharide biosynthetic process	1.52E-5	3.91E-3	49.61 (4118,3,83,3)	[+] Show genes
GO:0046390	ribose phosphate biosynthetic process	1.58E-5	3.97E-3	5.91 (4118,41,187,11)	[+] Show genes
GO:0051726	regulation of cell cycle	1.59E-5	3.9E-3	1.64 (4118,370,557,82)	[+] Show genes
GO:1902600	proton transmembrane transport	1.77E-5	4.24E-3	3.96 (4118,28,520,14)	[+] Show genes
GO:0044772	mitotic cell cycle phase transition	1.85E-5	4.35E-3	2.33 (4118,85,666,32)	[+] Show genes
GO:0022402	cell cycle process	1.87E-5	4.28E-3	1.80 (4118,307,454,61)	[+] Show genes
GO:0071241	cellular response to inorganic substance	1.94E-5	4.35E-3	2.97 (4118,42,661,20)	[+] Show genes
GO:0072522	purine-containing compound biosynthetic process	2.04E-5	4.48E-3	5.77 (4118,42,187,11)	[+] Show genes
GO:0008104	protein localization	2.47E-5	5.33E-3	1.75 (4118,579,264,65)	[+] Show genes
GO:0006163	purine nucleotide metabolic process	2.47E-5	5.22E-3	4.20 (4118,77,191,15)	[+] Show genes
GO:0002504	antigen processing and presentation of peptide or polysaccharide antigen via MHC class II	3.04E-5	6.29E-3	8.44 (4118,32,122,8)	[+] Show genes
GO:0002495	antigen processing and presentation of peptide antigen via MHC class II	3.04E-5	6.17E-3	8.44 (4118,32,122,8)	[+] Show genes
GO:0019886	antigen processing and presentation of exogenous peptide antigen via MHC class II	3.04E-5	6.05E-3	8.44 (4118,32,122,8)	[+] Show genes
GO:0007346	regulation of mitotic cell cycle	3.24E-5	6.33E-3	1.87 (4118,204,551,51)	[+] Show genes
GO:0072395	signal transduction involved in cell cycle checkpoint	3.34E-5	6.41E-3	3.61 (4118,25,638,14)	[+] Show genes
GO:0072422	signal transduction involved in DNA damage checkpoint	3.34E-5	6.29E-3	3.61 (4118,25,638,14)	[+] Show genes
GO:0072401	signal transduction involved in DNA integrity checkpoint	3.34E-5	6.18E-3	3.61 (4118,25,638,14)	[+] Show genes
GO:0033036	macromolecule localization	3.36E-5	6.11E-3	1.74 (4118,584,264,65)	[+] Show genes
GO:0009259	ribonucleotide metabolic process	3.46E-5	6.19E-3	4.09 (4118,79,191,15)	[+] Show genes
GO:0031023	microtubule organizing center organization	4.75E-5	8.35E-3	3.11 (4118,19,975,14)	[+] Show genes
GO:0006660	phosphatidylserine catabolic process	4.95E-5	8.57E-3	196.10 (4118,2,21,2)	[+] Show genes
GO:0044839	cell cycle G2/M phase transition	5.55E-5	9.45E-3	2.29 (4118,48,975,26)	[+] Show genes
GO:1901135	carbohydrate derivative metabolic process	5.78E-5	9.68E-3	2.62 (4118,191,206,25)	[+] Show genes
GO:0051056	regulation of small GTPase mediated signal transduction	5.96E-5	9.83E-3	3.95 (4118,96,163,15)	[+] Show genes
GO:0046461	neutral lipid catabolic process	6.11E-5	9.91E-3	62.39 (4118,9,22,3)	[+] Show genes
GO:0046464	acylglycerol catabolic process	6.11E-5	9.76E-3	62.39 (4118,9,22,3)	[+] Show genes
GO:0042770	signal transduction in response to DNA damage	6.13E-5	9.66E-3	2.98 (4118,39,638,18)	[+] Show genes
GO:0045851	pH reduction	6.4E-5	9.93E-3	13.40 (4118,8,192,5)	[+] Show genes
GO:0051452	intracellular pH reduction	6.4E-5	9.79E-3	13.40 (4118,8,192,5)	[+] Show genes
GO:1902903	regulation of supramolecular fiber organization	6.47E-5	9.76E-3	2.55 (4118,98,429,26)	[+] Show genes
GO:0006753	nucleoside phosphate metabolic process	6.75E-5	1E-2	3.37 (4118,115,191,18)	[+] Show genes
GO:0009117	nucleotide metabolic process	6.75E-5	9.89E-3	3.37 (4118,115,191,18)	[+] Show genes
GO:0006732	coenzyme metabolic process	6.96E-5	1.01E-2	12.91 (4118,66,29,6)	[+] Show genes
GO:0015985	energy coupled proton transport, down electrochemical gradient	7.34E-5	1.05E-2	17.62 (4118,5,187,4)	[+] Show genes
GO:0015986	ATP synthesis coupled proton transport	7.34E-5	1.03E-2	17.62 (4118,5,187,4)	[+] Show genes
GO:0042776	mitochondrial ATP synthesis coupled proton transport	7.34E-5	1.02E-2	17.62 (4118,5,187,4)	[+] Show genes
GO:0019693	ribose phosphate metabolic process	7.41E-5	1.02E-2	3.85 (4118,84,191,15)	[+] Show genes
GO:0006123	mitochondrial electron transport, cytochrome c to oxygen	7.46E-5	1.01E-2	8.62 (4118,5,478,5)	[+] Show genes
GO:0019646	aerobic electron transport chain	7.46E-5	9.96E-3	8.62 (4118,5,478,5)	[+] Show genes
GO:0043547	positive regulation of GTPase activity	8.29E-5	1.09E-2	3.65 (4118,108,167,16)	[+] Show genes
GO:0034308	primary alcohol metabolic process	8.62E-5	1.12E-2	15.53 (4118,17,78,5)	[+] Show genes
GO:0035338	long-chain fatty-acyl-CoA biosynthetic process	8.92E-5	1.15E-2	147.07 (4118,2,28,2)	[+] Show genes
GO:0035336	long-chain fatty-acyl-CoA metabolic process	8.92E-5	1.13E-2	147.07 (4118,2,28,2)	[+] Show genes
GO:0055085	transmembrane transport	8.98E-5	1.13E-2	6.70 (4118,273,18,8)	[+] Show genes
GO:0055086	nucleobase-containing small molecule metabolic process	1.04E-4	1.3E-2	3.15 (4118,130,191,19)	[+] Show genes
GO:0017144	drug metabolic process	1.12E-4	1.37E-2	3.39 (4118,108,191,17)	[+] Show genes
GO:0016024	CDP-diacylglycerol biosynthetic process	1.13E-4	1.37E-2	39.85 (4118,5,62,3)	[+] Show genes
GO:0046341	CDP-diacylglycerol metabolic process	1.13E-4	1.36E-2	39.85 (4118,5,62,3)	[+] Show genes
GO:0071156	regulation of cell cycle arrest	1.14E-4	1.35E-2	2.97 (4118,37,638,17)	[+] Show genes
GO:0009066	aspartate family amino acid metabolic process	1.22E-4	1.43E-2	24.01 (4118,14,49,4)	[+] Show genes
GO:0019637	organophosphate metabolic process	1.27E-4	1.47E-2	3.77 (4118,232,66,14)	[+] Show genes
GO:0044282	small molecule catabolic process	1.31E-4	1.51E-2	5.60 (4118,92,80,10)	[+] Show genes
GO:0010165	response to X-ray	1.42E-4	1.61E-2	5.17 (4118,8,697,7)	[+] Show genes
GO:0052651	monoacylglycerol catabolic process	1.44E-4	1.62E-2	130.73 (4118,3,21,2)	[+] Show genes
GO:0010564	regulation of cell cycle process	1.45E-4	1.61E-2	1.74 (4118,236,552,55)	[+] Show genes
GO:0071616	acyl-CoA biosynthetic process	1.53E-4	1.68E-2	47.33 (4118,9,29,3)	[+] Show genes
GO:0035384	thioester biosynthetic process	1.53E-4	1.66E-2	47.33 (4118,9,29,3)	[+] Show genes
GO:0061024	membrane organization	1.55E-4	1.67E-2	1.96 (4118,188,435,39)	[+] Show genes
GO:0006509	membrane protein ectodomain proteolysis	1.6E-4	1.7E-2	43.05 (4118,7,41,3)	[+] Show genes
GO:0070507	regulation of microtubule cytoskeleton organization	1.74E-4	1.84E-2	2.78 (4118,69,429,20)	[+] Show genes
GO:0033127	regulation of histone phosphorylation	1.85E-4	1.93E-2	11.10 (4118,4,371,4)	[+] Show genes
GO:1901568	fatty acid derivative metabolic process	1.91E-4	1.97E-2	22.88 (4118,24,30,4)	[+] Show genes
GO:0051088	PMA-inducible membrane protein ectodomain proteolysis	1.93E-4	1.98E-2	100.44 (4118,2,41,2)	[+] Show genes
GO:0009127	purine nucleoside monophosphate biosynthetic process	1.94E-4	1.97E-2	7.34 (4118,21,187,7)	[+] Show genes
GO:0009168	purine ribonucleoside monophosphate biosynthetic process	1.94E-4	1.95E-2	7.34 (4118,21,187,7)	[+] Show genes
GO:0044242	cellular lipid catabolic process	1.96E-4	1.95E-2	8.21 (4118,45,78,7)	[+] Show genes
GO:0046503	glycerolipid catabolic process	1.96E-4	1.93E-2	46.80 (4118,12,22,3)	[+] Show genes
GO:0006654	phosphatidic acid biosynthetic process	2.14E-4	2.09E-2	20.44 (4118,13,62,4)	[+] Show genes
GO:0046473	phosphatidic acid metabolic process	2.14E-4	2.07E-2	20.44 (4118,13,62,4)	[+] Show genes
GO:0043087	regulation of GTPase activity	2.38E-4	2.28E-2	3.22 (4118,130,167,17)	[+] Show genes
GO:0007088	regulation of mitotic nuclear division	2.42E-4	2.3E-2	3.26 (4118,46,412,15)	[+] Show genes
GO:0042984	regulation of amyloid precursor protein biosynthetic process	2.43E-4	2.29E-2	34.90 (4118,6,59,3)	[+] Show genes
GO:0000086	G2/M transition of mitotic cell cycle	2.71E-4	2.53E-2	2.20 (4118,46,975,24)	[+] Show genes
GO:0031334	positive regulation of protein complex assembly	2.71E-4	2.51E-2	1.92 (4118,79,949,35)	[+] Show genes
GO:0090383	phagosome acidification	2.89E-4	2.66E-2	14.30 (4118,6,192,4)	[+] Show genes
GO:1901992	positive regulation of mitotic cell cycle phase transition	2.96E-4	2.69E-2	3.60 (4118,27,508,12)	[+] Show genes
GO:0009062	fatty acid catabolic process	2.99E-4	2.7E-2	12.57 (4118,21,78,5)	[+] Show genes
GO:0051783	regulation of nuclear division	3.06E-4	2.73E-2	3.08 (4118,52,412,16)	[+] Show genes
GO:0060627	regulation of vesicle-mediated transport	3.09E-4	2.74E-2	2.20 (4118,132,426,30)	[+] Show genes
GO:1901566	organonitrogen compound biosynthetic process	3.43E-4	3.02E-2	3.63 (4118,238,62,13)	[+] Show genes
GO:0010519	negative regulation of phospholipase activity	3.47E-4	3.03E-2	17.52 (4118,3,235,3)	[+] Show genes
GO:0051128	regulation of cellular component organization	3.48E-4	3.01E-2	1.44 (4118,678,434,103)	[+] Show genes
GO:0002084	protein depalmitoylation	3.67E-4	3.15E-2	102.95 (4118,4,20,2)	[+] Show genes
GO:2000045	regulation of G1/S transition of mitotic cell cycle	3.73E-4	3.18E-2	2.46 (4118,54,651,21)	[+] Show genes
GO:0045787	positive regulation of cell cycle	3.84E-4	3.24E-2	1.94 (4118,118,666,37)	[+] Show genes
GO:1902400	intracellular signal transduction involved in G1 DNA damage checkpoint	3.96E-4	3.31E-2	3.37 (4118,23,638,12)	[+] Show genes
GO:1902403	signal transduction involved in mitotic DNA integrity checkpoint	3.96E-4	3.29E-2	3.37 (4118,23,638,12)	[+] Show genes
GO:1902402	signal transduction involved in mitotic DNA damage checkpoint	3.96E-4	3.26E-2	3.37 (4118,23,638,12)	[+] Show genes
GO:0006977	DNA damage response, signal transduction by p53 class mediator resulting in cell cycle arrest	3.96E-4	3.24E-2	3.37 (4118,23,638,12)	[+] Show genes
GO:0072431	signal transduction involved in mitotic G1 DNA damage checkpoint	3.96E-4	3.21E-2	3.37 (4118,23,638,12)	[+] Show genes
GO:0072413	signal transduction involved in mitotic cell cycle checkpoint	3.96E-4	3.19E-2	3.37 (4118,23,638,12)	[+] Show genes
GO:0030330	DNA damage response, signal transduction by p53 class mediator	3.98E-4	3.19E-2	2.93 (4118,33,638,15)	[+] Show genes
GO:0046462	monoacylglycerol metabolic process	4.06E-4	3.22E-2	98.05 (4118,4,21,2)	[+] Show genes
GO:0034033	purine nucleoside bisphosphate biosynthetic process	4.11E-4	3.24E-2	35.50 (4118,12,29,3)	[+] Show genes
GO:0034030	ribonucleoside bisphosphate biosynthetic process	4.11E-4	3.22E-2	35.50 (4118,12,29,3)	[+] Show genes
GO:0033866	nucleoside bisphosphate biosynthetic process	4.11E-4	3.19E-2	35.50 (4118,12,29,3)	[+] Show genes
GO:0072425	signal transduction involved in G2 DNA damage checkpoint	4.14E-4	3.19E-2	16.60 (4118,3,248,3)	[+] Show genes
GO:0033047	regulation of mitotic sister chromatid segregation	4.19E-4	3.2E-2	5.51 (4118,23,260,8)	[+] Show genes
GO:0006119	oxidative phosphorylation	4.41E-4	3.35E-2	5.94 (4118,8,520,6)	[+] Show genes
GO:1901570	fatty acid derivative biosynthetic process	4.48E-4	3.38E-2	34.32 (4118,12,30,3)	[+] Show genes
GO:0042181	ketone biosynthetic process	4.71E-4	3.52E-2	8.74 (4118,4,471,4)	[+] Show genes
GO:2000134	negative regulation of G1/S transition of mitotic cell cycle	4.74E-4	3.52E-2	2.79 (4118,37,638,16)	[+] Show genes
GO:0110053	regulation of actin filament organization	4.84E-4	3.57E-2	3.96 (4118,77,162,12)	[+] Show genes
GO:0048870	cell motility	4.97E-4	3.64E-2	1.91 (4118,202,395,37)	[+] Show genes
GO:0090068	positive regulation of cell cycle process	5.03E-4	3.66E-2	2.13 (4118,83,651,28)	[+] Show genes
GO:0032886	regulation of microtubule-based process	5.13E-4	3.71E-2	2.59 (4118,74,429,20)	[+] Show genes
GO:1901989	positive regulation of cell cycle phase transition	5.18E-4	3.72E-2	3.23 (4118,30,552,13)	[+] Show genes
GO:0009126	purine nucleoside monophosphate metabolic process	5.21E-4	3.71E-2	4.98 (4118,39,191,9)	[+] Show genes
GO:0009167	purine ribonucleoside monophosphate metabolic process	5.21E-4	3.69E-2	4.98 (4118,39,191,9)	[+] Show genes
GO:0042159	lipoprotein catabolic process	5.28E-4	3.71E-2	13.00 (4118,7,181,4)	[+] Show genes
GO:0009156	ribonucleoside monophosphate biosynthetic process	5.52E-4	3.86E-2	6.42 (4118,24,187,7)	[+] Show genes
GO:1901661	quinone metabolic process	5.53E-4	3.84E-2	8.42 (4118,4,489,4)	[+] Show genes
GO:1901987	regulation of cell cycle phase transition	5.55E-4	3.83E-2	1.84 (4118,141,651,41)	[+] Show genes
GO:0042219	cellular modified amino acid catabolic process	5.7E-4	3.91E-2	26.06 (4118,6,79,3)	[+] Show genes
GO:0071158	positive regulation of cell cycle arrest	5.77E-4	3.93E-2	3.11 (4118,27,638,13)	[+] Show genes
GO:0072329	monocarboxylic acid catabolic process	5.85E-4	3.95E-2	11.00 (4118,24,78,5)	[+] Show genes
GO:0006081	cellular aldehyde metabolic process	6.14E-4	4.13E-2	10.86 (4118,24,79,5)	[+] Show genes
GO:0006638	neutral lipid metabolic process	6.15E-4	4.11E-2	33.03 (4118,17,22,3)	[+] Show genes
GO:0006639	acylglycerol metabolic process	6.15E-4	4.08E-2	33.03 (4118,17,22,3)	[+] Show genes
GO:0090503	RNA phosphodiester bond hydrolysis, exonucleolytic	6.27E-4	4.14E-2	3.33 (4118,19,717,11)	[+] Show genes
GO:0010035	response to inorganic substance	6.33E-4	4.15E-2	1.88 (4118,126,661,38)	[+] Show genes
GO:0055114	oxidation-reduction process	6.46E-4	4.21E-2	3.08 (4118,233,86,15)	[+] Show genes
GO:0034314	Arp2/3 complex-mediated actin nucleation	6.52E-4	4.22E-2	19.06 (4118,4,162,3)	[+] Show genes
GO:1902807	negative regulation of cell cycle G1/S phase transition	6.87E-4	4.42E-2	2.72 (4118,38,638,16)	[+] Show genes
GO:0070925	organelle assembly	7.01E-4	4.48E-2	2.05 (4118,151,426,32)	[+] Show genes
GO:1902806	regulation of cell cycle G1/S phase transition	7.09E-4	4.51E-2	2.32 (4118,60,651,22)	[+] Show genes
GO:0043536	positive regulation of blood vessel endothelial cell migration	7.19E-4	4.54E-2	9.95 (4118,18,115,5)	[+] Show genes
GO:0006307	DNA dealkylation involved in DNA repair	7.28E-4	4.57E-2	18.28 (4118,4,169,3)	[+] Show genes
GO:0006996	organelle organization	7.44E-4	4.64E-2	1.42 (4118,711,412,101)	[+] Show genes
GO:0007026	negative regulation of microtubule depolymerization	7.62E-4	4.73E-2	5.21 (4118,13,426,7)	[+] Show genes
GO:1905818	regulation of chromosome separation	7.66E-4	4.72E-2	4.06 (4118,18,507,9)	[+] Show genes
GO:0042981	regulation of apoptotic process	7.69E-4	4.72E-2	1.79 (4118,406,250,44)	[+] Show genes
GO:0042886	amide transport	8.56E-4	5.22E-2	1.89 (4118,419,192,37)	[+] Show genes
GO:0046475	glycerophospholipid catabolic process	8.91E-4	5.4E-2	78.44 (4118,5,21,2)	[+] Show genes
GO:0042574	retinal metabolic process	9.07E-4	5.47E-2	22.92 (4118,7,77,3)	[+] Show genes
GO:0097305	response to alcohol	9.36E-4	5.61E-2	2.12 (4118,48,929,23)	[+] Show genes
GO:0009161	ribonucleoside monophosphate metabolic process	9.47E-4	5.64E-2	4.62 (4118,42,191,9)	[+] Show genes
GO:0043603	cellular amide metabolic process	9.58E-4	5.68E-2	6.58 (4118,151,29,7)	[+] Show genes
GO:0006554	lysine catabolic process	9.71E-4	5.73E-2	1,029.50 (4118,1,4,1)	[+] Show genes
GO:0006569	tryptophan catabolic process	9.71E-4	5.69E-2	1,029.50 (4118,1,4,1)	[+] Show genes
GO:0006568	tryptophan metabolic process	9.71E-4	5.66E-2	1,029.50 (4118,1,4,1)	[+] Show genes
GO:0006586	indolalkylamine metabolic process	9.71E-4	5.63E-2	1,029.50 (4118,1,4,1)	[+] Show genes
GO:0019474	L-lysine catabolic process to acetyl-CoA	9.71E-4	5.6E-2	1,029.50 (4118,1,4,1)	[+] Show genes
GO:0019477	L-lysine catabolic process	9.71E-4	5.57E-2	1,029.50 (4118,1,4,1)	[+] Show genes
GO:0033512	L-lysine catabolic process to acetyl-CoA via saccharopine	9.71E-4	5.54E-2	1,029.50 (4118,1,4,1)	[+] Show genes
GO:0042436	indole-containing compound catabolic process	9.71E-4	5.51E-2	1,029.50 (4118,1,4,1)	[+] Show genes
GO:0046218	indolalkylamine catabolic process	9.71E-4	5.48E-2	1,029.50 (4118,1,4,1)	[+] Show genes
GO:0046440	L-lysine metabolic process	9.71E-4	5.45E-2	1,029.50 (4118,1,4,1)	[+] Show genes
GO:0052652	cyclic purine nucleotide metabolic process	9.79E-4	5.47E-2	43.81 (4118,2,94,2)	[+] Show genes
GO:0009190	cyclic nucleotide biosynthetic process	9.79E-4	5.44E-2	43.81 (4118,2,94,2)	[+] Show genes

Species used: Homo sapiens

The system has recognized 5652 genes out of 5850 gene terms entered by the user.
5652 genes were recognized by gene symbol and 0 genes by other gene IDs .
1364 duplicate genes were removed (keeping the highest ranking instance of each gene) leaving a total of 4288 genes.
Only 4118 of these genes are associated with a GO term.

The GOrilla database is periodically updated using the GO database and other sources.
The GOrilla database was last updated on Oct 17, 2020

This results page will be available on this site for one month from now (until Nov 22, 2020). You can bookmark this page and come back to it later.

'P-value' is the enrichment p-value computed according to the mHG or HG model. This p-value is not corrected for multiple testing of 10552 GO terms.

'FDR q-value' is the correction of the above p-value for multiple testing using the Benjamini and Hochberg (1995) method.
Namely, for the ith term (ranked according to p-value) the FDR q-value is (p-value * number of GO terms) / i.

Enrichment (N, B, n, b) is defined as follows:
N - is the total number of genes
B - is the total number of genes associated with a specific GO term
n - is the number of genes in the top of the user's input list or in the target set when appropriate
b - is the number of genes in the intersection
Enrichment = (b/n) / (B/N)

Genes: For each GO term you can see the list of associated genes that appear in the optimal top of the list.
Each gene name is specified by gene symbol followed by a short description of the gene

Back to the GOrilla main page
